# Adapting a consensus process for survivors of domestic abuse and child maltreatment: a brief report about adopting a trauma-informed approach in multistakeholder workshops

**DOI:** 10.1136/bmjopen-2024-090017

**Published:** 2025-01-22

**Authors:** Claire Powell, Eszter Szilassy, Katherine Cowan, Gene Feder, Ruth Gilbert, Emma Howarth, Karen Johns, Ursula Lindenberg, Alison Gregory

**Affiliations:** 1 Institute of Child Health, University College London, London, UK; 2 Centre for Academic Primary Care, Population Health Sciences, Bristol Medical School, University of Bristol, Bristol, UK; 3 Katherine Cowan Consulting Limited, St. Leonards-on-Sea, UK; 4 James Lind Alliance, Southampton, UK; 5 Community Based Medicine, University of Bristol, Bristol, UK; 6 Centre for Paediatric Epidemiology and Biostatistics, UCL Institute of Child Health, London, UK; 7 Psychology, University of Sussex, Brighton, UK; 8 Voices Charity, Bath, UK; 9 Alison Gregory Consultancy, Bristol, UK

**Keywords:** Community Participation, Delphi Technique, Health, Methods, PUBLIC HEALTH

## Abstract

**Abstract:**

**Purpose:**

Among health researchers, there is a growing appreciation of the importance of the involvement of service users and members of the public. This recognition has not only resulted in involvement guidelines and improved research ethics but also an increasing use of consensus processes with service users and members of the public to determine research priorities and questions and to agree outcomes to be measured in intervention studies. There is, however, limited advice about how to safely involve survivors of violence and abuse in consensus-based studies.

**Methods/results:**

This commentary provides an overview of the adaptations made to a process of core outcome set development, to ensure that survivors of violence and abuse felt safe, heard and supported, and able to contribute in a meaningful way.

**Conclusions:**

We advocate for an iterative process of listening to and learning from survivors, as well as buy-in from funders to ensure research studies are appropriately resourced and involve sufficient planning time.

## Background

 Public involvement in health research—involving health service users and their carers in the design and delivery of research—is widely accepted in the UK as positive and is increasingly a requirement in funding applications. For example, the National Institute for Health and Care Research (NIHR)—one of the main health research funders in the UK—has developed the UK Standards for Public Involvement^1^ to improve research practice. Alongside this, there is growing use of priority setting^2 3^ and consensus processes^4 5^ which enable service users to play a role in determining research priorities and questions, developing outcome sets and more actively influencing health research agendas. Guidelines to formalise pay and remuneration and ensure accessibility, diversity and inclusion have also been developed.^16^,^8^ However, criticisms remain about researchers’ tokenistic approaches to involvement^9^ and few people report when public involvement goes wrong.^10^

While much of the guidance, for what is termed in the UK Patient and Public Involvement, is focused on accessibility from a disability perspective (eg, providing materials that are accessible to children or adults with learning disabilities, or with hearing and sight impairments), involving survivors of violence and abuse in research processes necessitates nuanced, trauma-informed thinking.^11^ We understood trauma-informed to mean avoiding retraumatising participants and prioritising safety and trust.^12^

Since the WHO guidelines in 2005,^13^ there is a growing literature on how to conduct research respectfully and safely with survivors of violence and abuse while also protecting researcher well-being.^14^,^17^ However, much of this is focused on individual interviews, or analysis and dissemination.^18 19^ Much less thought has been given to survivor participation in other forms of applied research that may bring people into contact with a range of stakeholders in workshops or consensus group meetings, both in general, and more particularly, in the postpandemic virtual context. Even comprehensive guides, such as the researcher integrity framework,^20^ do not explicitly mention multistakeholder groups.

Involving survivors in multistakeholder groups alongside researchers and practitioners requires reflection and planning that extends beyond current guidance. In such settings, survivors may find themselves in a position where they need to interact with professional groups which they feel have been harmful or retraumatising in their care. Survivors’ motivations to take part in multistakeholder research may also be rooted in former interactions with professional groups that were experienced as harmful to themselves and others,^21^ and they want to contribute to research that could involve systems learning and improvement. Further, if contention and conflict are not managed sensitively by facilitators, the process of negotiation to reach a consensus may itself be triggering or retraumatising. The scope to cause harm in the research process needs careful consideration, in much the same way that services for survivors of violence and abuse need to work in ways that avoid retraumatisation.^12^

This commentary describes and reflects on the methods used to adapt multistakeholder workshops, as part of a consensus process in the context of core outcome development, so that workshops were more trauma-informed, jointly owned and collaborative while minimising the pitfall of othering narratives and actions. Here, we describe a process of iterative learning from survivor feedback, to increase inclusivity and to reduce potential harms. Our findings and reflections are applicable to any aspects of research that involve multistakeholder meetings or workshops, including consultation, coproduction, participatory research and consensus processes.

## Research aim

The multistakeholder workshops, on which this paper reflects, were components of a larger consensus study to develop core outcome sets (COSs) for the evaluation of interventions for children and families with experience of, or at risk of, child maltreatment (CM) or domestic violence and abuse (DVA).^22^ COSs are a minimum set of outcomes reported in trials, or other types of research and evaluation, to reduce research wastage and improve the reporting consistency, so that interventions can be better compared.^5^ Improving comparability is important to survivors receiving services because it can support improvement, best practice and lessen the likelihood of receiving uneven services and encountering varying approaches to support around the country.^23^

## Methods

We registered our study and published a research protocol.^24^ The multimethod process took part in two stages: (1) identifying candidate outcomes from 6 evidence reviews, 2 stakeholder workshops and 10 qualitative interviews (we defined an ‘outcome’ as something that could be measured - whether a tool to measure it currently exists or not - to assess intervention effectiveness, e.g., an outcome could be a measure of emotional well-being, or community cohesion, or family relationships.) ; (2) reaching consensus on final COSs through two e-Delphi surveys and two consensus workshops. (A Delphi survey is a structured approach to reaching a consensus between panel members.) In this paper, we focus on the learning from the initial stakeholder workshops in stage 1 and the improvements in our approach for the consensus workshops in stage 2. We have reported these reflections as a commentary because we did not formally collect data related to the process of workshop delivery and what follows reflects discussion and learning from the research team and survivor advisory group.

### Participants

We recruited from the following groups for both the initial and the consensus workshops: (a) UK-based adults with personal experience of CM/DVA or experience as the parent of a child who had experienced CM/DVA; (b) UK-based practitioners delivering, managing or commissioning CM/DVA services and (c) English-speaking CM/DVA researchers based in universities or research organisations in high-income countries. We did not directly involve children and young people aged under 18 years because of the potential safeguarding issues if they had recent experiences of violence and abuse and were not engaged with support services.

The stakeholder workshops were advertised in the first stage of the project through CM/DVA specialist organisations, university-based lived experience networks, the Violence, Abuse and Mental Health Network and the National Survivor User Network. In the second stage of the project, participants in the e-Delphi survey were asked to express an interest in the subsequent workshops and we invited people in a random order until the spaces were filled.

## Study process and reflections

### A: initial multistakeholder workshops

The initial stakeholder workshops were held in person at the beginning of the COS development process. The aim was to bring together survivors, practitioners and researchers to identify, discuss and define outcomes that could be used to measure the impact of services and support. The workshops involved presentations from external research experts on CM and DVA research, in addition to an overview of the project aims from the team. Participants were arranged around tables, with volunteer note-takers who also acted as facilitators when required. These volunteers were people known to the research team and were given brief instructions prior to the workshop.

In planning the workshops, advice was sought from survivor researchers in the team’s network, with a focus on the number of survivors taking part. Our aim was to recruit equal numbers of survivors and researchers/practitioners and to pair survivors within small discussion groups, so they were never the sole voice of lived experience in their groups. Both in the lead-up to the workshop and immediately afterwards, the research team had contact with individual survivors. Payments in excess of NIHR guidelines were offered, in recognition of the depth and impact of the information people were being asked to share.

While the primary aims of the workshops were successfully met, there were major challenges to the process. Survivor feedback indicated that some participants who were attending in a professional capacity used alienating language and dismissive behaviour when discussing research. Some participants tried to impose their own theories on survivors’ experiences and prioritised their professional knowledge over knowledge gained by lived experience. Some academic researchers were publicly combative (in debating contrasting views to defend their professional stance), resulting in survivors feeling that academic sparring overshadowed debates, with the voice of survivor insight being undermined. It was triggering for some survivors when dialogue was shut down or overridden; they felt as though they were not listened to, understood or heard. These challenges were compounded in the first workshop by the size of the participant group (over 50 participants) which made it difficult for the research team to be sufficiently available to survivors and to intervene effectively.

On reflection, the research team recognised that while focusing on survivor representation and inclusion, we had omitted consideration of the behaviour and impact of attending practitioners and researchers. Refocusing from inclusion to ‘maximising opportunities for positive experiences’^25^ for survivors and more thoughtful consideration of the experience of the workshops would have helped us to plan more carefully. Although the workshop advertising explained that the group included survivors, there was no explanation about the implications for, and expectations of, researchers and practitioners. Although many domestic abuse practitioners are also survivors, we still needed clear guidelines to ensure the space was trauma informed.

The team’s reflections resonated with survivor feedback which highlighted the following: (1) researchers and practitioners need clear guidelines or terms of reference about how to behave and to carefully communicate with survivors in multistakeholder workshops; (2) high conflict academic debate, while stimulating for some, can be triggering for survivors of violence and abuse and needs to be avoided in workshop settings and (3) a mutual understanding is needed about survivors’ voices being held in the same regard as the voice of researchers and practitioners, with a sense that everyone is working towards the same goal.

Because of the study timeline, the formation of lived experience advisory groups had been delayed until after the stage 1 stakeholder workshops. On reflection, the team recognised that these advisory groups should have been formed much earlier in the research process to input into the workshop planning. Having recognised our error, we engaged two groups of survivors to advise on all subsequent aspects of study design.

### B: consensus workshops

By the time the final stage of the consensus process was reached, much of our approach had been modified so that it was underpinned by trauma-informed principles and practices to ensure safety, build trust and avoid retraumatisation. We revised our use of language and incorporated a longer lead-in time to plan survivor involvement in the final round of workshops. It was particularly important that the consensus workshops were well thought out because the groups were smaller (a maximum of 24 people) with equal numbers of (1) survivors, (2) researchers and (3) practitioners. We drew on the James Lind Alliance (JLA) method, which is designed to involve multiple stakeholder groups equally, and which itself draws on nominal group technique,^26^ a structured approach ensuring every participant has equal opportunities to participate by speaking in turn for set amounts of time. In addition, the COVID-19 pandemic altered our ability to meet in person, and the workshops moved to being entirely online. This had some positive repercussions because it enabled us to involve participants from across the country, including people who were unable to travel, though it also meant we were unable to include participants without access to the internet.

Our survivor advisory groups, having reviewed the study materials and processes to ensure clarity, asked for two specific changes: (1) that a counsellor (AG) was provided throughout the process to provide support and debriefing and (2) that participants were given advance information about attendees, so that survivors were not caught unawares by the individual practitioners or the organisations they represented. This was in part to safeguard survivors on the advice of our lived experience groups, for example, those who could still be at risk for disclosing experiences if they were remaining in or at risk of a return to the Family Court system and could not participate if practitioners, for example, social workers were in the workshop.

An external independent facilitator (KC) was also employed. The facilitator had specific expertise in priority setting with consensus methods, involvement of seldom heard groups and experience in delivering JLA priority setting workshops.^2^ The decision to employ an independent facilitator was undertaken for two reasons: first, an external facilitator was considered more neutral/independent than the research team, and second, we wanted to bring in expertise around consensus methods and the involvement of diverse stakeholder perspectives, including online facilitation.

In terms of planning the workshops, a much greater amount of time (4 months) was given than for our initial stakeholder workshops. The research team, in partnership with KC, constructed detailed workshop plans with regular breaks, ensuring structured consensus discussions. Again, drawing on the JLA approach,^2^ the method was underpinned by the principle of equal involvement of different stakeholder groups and used mixed breakout groups, to provide opportunities for participants to share their perspectives and learn from one other in smaller and thus less intimidating groups. Unlike JLA workshops, our aim was not to rank all items (outcomes) to determine a top 10 with consensus reached through dialogue alone. Instead, participants were asked to discuss the outcomes and then vote, to work out a shortlist of five core outcomes—more akin to an adapted Delphi meeting approach.

The following was developed to guide the conduct of the workshops: (a) a set of trauma-informed principles that all participants were required to sign before attending, emphasising the equal value of knowledge based on lived experience and knowledge based on professional expertise (these principles were further reiterated during the introductory presentation for the workshops); (b) brief self-descriptions by participants, shared in advance of the workshops, for transparency; (c) clear document packs explaining the workshop plan, how to record decisions and how voting worked (shared in advance) and (d) an agreed protocol about names, confidentiality and optional use of online cameras (shared in advance and discussed on the day). In addition, the facilitators of small group discussions were notified of the survivors in their group so they could offer additional support, and counselling/debriefing support was made available for individuals throughout the workshops, and then as a group after the workshop was complete.

In advance of the workshop, we carried out a full briefing with facilitators to discuss the process, neutrality, inclusive facilitation techniques and safeguarding. Managing paternalistic/dominant conduct and the use of insensitive language were also discussed. To ensure safety and confidentiality, survivors were advised that they could use pseudonyms; researchers and practitioners were required to share their first name (as a minimum) and their organisation (so that survivors were aware of who would be attending). An advanced technical rehearsal was carried out to ensure the smooth running of the online platform, which included a run-through of the practical process for connecting a survivor with the counsellor, if requested. Survivors unfamiliar with the online platform (Zoom) were also offered a premeet to answer questions and offer technical support.

During the workshop, the facilitation team met in a virtual breakout room during breaks to debrief. These conversations included discussions about participants’ welfare and were an opportunity to flag any concerns or potential requests to connect with the counsellor. While the groups were reconfigured over two rounds of discussion, survivors remained with the same facilitator, to maximise the opportunity to build rapport and to understand people’s support needs. In the final plenary, which brought everyone together, people had the option to speak, use the chat or contribute anonymously on an online whiteboard. This was to ensure the discussion was accessible for people who might be uncomfortable contributing to a larger group or who had things they wanted to say anonymously.

The feedback from the consensus workshops suggested these strategies, in addition to the structure of the nominal group technique, were successful in creating a safe and accessible environment for survivors to take part. Survivors reported feeling able to speak in front of practitioners and researchers, some for the first time. Additionally, within the small group discussion, both practitioners and researchers shared lived experiences; this did not happen in the initial stakeholder workshops. We felt this to be an important step forward, not least in demonstrating the development of trust and a reduction in the divide that can happen between practitioners/researchers and survivors. After the consensus workshops, further access to the counsellor was requested, which we were able to make available. Initially, this support was offered solely to participants who had identified as survivors (because of uncertainty about uptake and capacity); subsequently, the offer was opened to all participants in recognition of the prevalence of lived experience among those working in the CM and DVA sectors. Although no one chose to access the counselling provided, survivor feedback nonetheless indicated that the availability of the counselling made them feel reassured that help would be available should they need it following the sessions. An important challenge was flagged, however, about how difficult it can be to confide in an unknown counsellor, in particular for survivors of DVA with concerns about the use of information as evidence in the family court system. Our survivor advisory group offered debriefing to their clients who knew each other, and these participants were able to connect after the meeting in a facilitated setting to support one another.

## Discussion

Traditional ways of involving service users in research, particularly in consensus processes, need to be extended if research processes are to be accessible to, and inclusive of, survivors of trauma. It is important to consider how elements of the research process can be potentially retraumatising or cause harm to survivors. This report reflects our learning through a 2-year multimethod, multistakeholder study and indicates the adaptations made to the study design to reflect survivor feedback and team observations on the process.

The key learning when designing and delivering consensus processes can be summarised as follows:

### Meaningful survivor involvement

Survivor involvement needs to be incorporated from the project planning stage and throughout consensus processes. This involvement includes advising on the language, accessibility and trauma-informed nature of materials, venue, location, events, format and researcher behaviour. To be meaningful, survivor consultation needs to be sustained and suggestions implemented. This involves sharing purpose, ownership and control of research, so that everyone feels like they are an equal partner (expert) in the process (coproduction: everyone is of equal importance, reciprocity, horizontal and more equitable relationships). There must not be them and us approach, so researchers need a nuanced understanding of power dynamics and be equipped to manage those that arise in groups, being proactive in promoting inclusion.

### Adopting a trauma-informed approach

The importance of adopting a trauma-informed approach to research involving survivors of violence and abuse cannot be overstated. Getting it right meant drawing heavily on expertise external to our team, even though we were a group of experienced researchers. Only with repeated consultation with survivors and an active responsiveness to their feedback were we able to anticipate needs and create a safe environment. This included access to specialist counselling and peer support, and careful planning of the multistakeholder workshop to ensure all participants interacted sensitively. We found clear, shared terms of reference, ground roles and responsibilities to be key, along with briefing and debriefing for the facilitators and an external, experienced facilitator. For online workshops, careful thought is needed around safety, particularly regarding the presence/absence of unseen people who are present but off-camera, which can compromise the transparency of workshops for DVA survivors. Hybrid workshops bring similar challenges in addition to supporting in-person attendees. Face-to-face workshops can enable more sensitive support for survivors, and greater security regarding the known presence of attendees.

### Commitment to accessibility and inclusion

While unable to accommodate everyone’s needs for the consensus workshops due to the requirement to hold the workshops online, allowing extra time and having funds to provide dedicated support meant that we could step beyond a vague commitment to accessibility and respond to the specific and articulated needs of individuals. If research processes, by lack of accessibility, effectively exclude participants and their contributions, the findings will be partial at best. If the same populations and communities are continually excluded due to access challenges, participation in research becomes inequitable and the validity of findings questionable. Trauma-informed accessibility is crucial to enable everyone to participate and for the organisers to address the needs of individuals who may represent groups who are most underserved. We held final plenary sessions in each consensus workshop to discuss how the COS might be inadvertently excluding minoritised survivors. However, a proactive prospective approach is needed to ensure representative and appropriate inclusion of survivors from seldom-heard groups.^27^

### Responsiveness and ongoing dialogue

Key to the success of the second round of workshops in terms of accessibility and acceptability was an ongoing dialogue with survivors, and an active responsiveness to their feedback and suggestions. This involved responding flexibly to feedback throughout the consensus process and changing, for example, survey wording when participants highlighted a lack of clarity. In addition, processes to gather and share updates with participants, and actions taken due to feedback were necessary, as was continuous reflection on learning from previous work/stages. This involved honest discussion of challenges and negative feedback, with a genuine commitment to improvement.

While there is a growing body of survivor-led trauma-informed literature,^28^ there is sparse literature regarding the conduct of multistakeholder workshops and research processes with survivors. Moreover, standard university research ethics processes are ill equipped to support adaption of standard research approaches to meet the needs of particular populations or methodological approaches.^29^,^34^ Paternalistic approaches to research ethics such as judging participants as ‘too vulnerable’ to take part can prevent research with people who have experienced trauma; this in turn limits our knowledge of violence and abuse.^33^ Topics that research ethics committees deem to be too sensitive are not regarded as such by research participants,^32^ highlighting the importance of lived experience involvement throughout review processes. In addition, committees are less likely to consider the impact on researchers working on sensitive topics.^29 34^ Finally, it has been repeatedly shown that research ethics committees are inconsistent in their requirements and judgements adding to confusion for researchers.^30^,^32^

However, recent work conducted since our workshops suggests that there is a growing interest in this area. There have been developments in critiquing and expanding university ethics boards,^35^ in addition to reports of trauma-informed multistakeholder consensus workshops involving survivor advisors and peer researchers,^36^ and survivors and practitioners with lived experience.^37^

## Conclusion

In conclusion, relying on standard approaches to access and involvement in research is insufficient to ensure consensus processes are inclusive and do not retraumatise survivors of abuse and violence. As researchers, it is important we do not avoid involving survivors because of our concerns, however, it is crucial that we plan and design research studies that are not harmful and that are positive and empowering experiences. This requires an iterative process of listening to and learning from survivors, as well as buy-in from funders to ensure research studies are appropriately resourced and involve sufficient planning time.

Ultimately, a trauma-informed approach to research involving lived experience participation is beneficial for everyone involved in this endeavour and should not only be a concern for those who have suffered the kinds of trauma being researched. There is much to gain both for the survivors of violence and abuse, who may be otherwise further harmed, marginalised and silenced and for the professional communities among whom there are also significant levels of the lived experience of trauma.
