# Supplementary material for: Adapting a consensus process for survivors of domestic abuse and child maltreatment: a brief report about adopting a trauma-informed approach in multistakeholder workshops
Source: BMJ Open. 2025 Jan 22;15(1):e090017. doi: 10.1136/bmjopen-2024-090017 (PMC11784130; doi:10.1136/bmjopen-2024-090017)
Supplement: Supplementary file 1 [file bmjopen-2024-090017.reviewer_comments.pdf]

PEER REVIEW HISTORY

BMJ Open publishes all reviews undertaken for accepted manuscripts. Reviewers are asked to complete a checklist review form and are provided with free text boxes to elaborate on their assessment. These free text comments are reproduced below.

ARTICLE DETAILS

Title (Provisional)

Adapting a consensus process for survivors of domestic abuse and child maltreatment: A brief report about adopting a trauma-informed approach in multi-stakeholder workshops.

Authors

Powell, Claire; Szilassy, Eszter; Cowan, Katherine; Feder, Gene; Gilbert, Ruth; howarth, emma; Johns, Karen; Lindenberg, Ursula; Gregory, Alison

---

VERSION 1 - REVIEW

**Reviewer**	**1**
**Name**	**Bandyopadhyay, Siddhartha**
**Affiliation**	**University of Birmingham, The Department of Economics**
**Date**	**13-Oct-2024**
**COI**	

It was been a pleasure to read this important piece dealing with how to ensure survivors of violence/maltreatment can participate in a consensus workshop with other stakeholders (where there is the possibility of facing conflict and the risk of re-traumatization) in a safe manner while contributing to the discussion in a meaningful way. It is refreshing to see learning from the initial workshop, clearly indicating problems that needed to be addressed, being discussed in a candid way.

The point around 'standard university research ethics processes are ill-equipped to support adaption of standard research approaches to meet the needs of particular populations' is well taken. If possible, within the word limit, some elaboration of this would make this even more useful for the reader.

It would also be useful for researchers planning workshops to keep in mind advantages and disadvantages when considering whether online forums or offline forums are most appropriate as well as consider hybrid formats while recognising the challenge of organising them and engaging both online and offline participants simultaneously.

**Reviewer**	**2**
**Name**	**Otterman, Gabriel**
**Affiliation**	**Linköping University, Department of Biomedical and Clinical Sciences**
**Date**	**28-Oct-2024**
**COI**	

Many thanks for the invitation to review this manuscript. The authors present a thoughtfully written account of challenges encountered during the conduct of a multi-phase consensus study with the overall aim of developing core outcome sets to enhance interventions for family violence and abuse. In the current paper, the investigators offer a reflective commentary on the challenges and adaptations made to foster a trauma-informed environment in multi-stakeholder workshops that included adults with lived experience of CM and DVA, alongside researchers and practitioners.

The manuscript is really well written, and the reflections are engaging. The authors make a compelling argument that standard approaches to public involvement must be adapted when conducting research in the intersecting and complex areas of CM and DVA. Their emphasis on ensuring that studies are not only safe but also positive and empowering for participants with lived experience underscores the need for innovative and iteratively refined methods. Funders and decision-makers should be made aware of the complexities and challenges inherent in this approach so that such research is adequately resourced, enabling genuine inclusion that moves beyond tokenism.

Without having read an original submission or previous reviewer comments, if any, I offer a few suggestions to strengthen the manuscript for publication.

Instead of presenting a formal thematic analysis, the authors provide informal reflections on the workshop process. While these reflections are insightful, a more structured qualitative analysis of the workshop data, as indicated in the previously published protocol (Howarth et al. 2022), would balance descriptions of what worked well with the challenges encountered. The protocol indicates a thematic analysis using Braun and Clarke; however, this analysis is not fully realised in the current commentary. A formal analysis could identify clear themes in participants' feedback, providing readers with a more complete understanding of the workshop dynamics. Further, incorporating quotes from participants would bring readers closer to the authentic voices of those involved. If the choice to report reflections in a commentary rather than perform thematic analysis was due to logistical constraints or other reasons, an explanation in the methods would provide important context for readers.

The manuscript focuses more on the challenges encountered in the conduct of the workshop and the subsequent pivot to implementing a more trauma-informed approach. A

more complete analysis might have revealed some of the successful elements alongside difficulties and would present a comprehensive view of the adaptations.

Providing demographic data, such as age, gender, ethnicity, lived experience affiliation, and LGBTQI status of workshop participants, researchers and practitioners, would offer deeper context for understanding the diversity of participant perspectives. A demographic summary table would enhance the analysis of trauma-informed adaptations.

A more detailed description of how "trauma-informed" was defined and operationalised would be helpful. The authors could discuss how they arrived at addressing specific behaviours, facilitation methods, and written guidelines for participant interaction that were subsequently implemented.

The manuscript does not appear to include a specific explanation for the lack of inclusion of children <18 in the workshops, which was well-delineated in the published protocol.

VERSION 1 - AUTHOR RESPONSE

**Reviewer 1**	
1. It was a pleasure to read this important piece dealing with how to ensure survivors of violence/maltreatment can participate in a consensus workshop with other stakeholders (where there is the possibility of facing conflict and the risk of re-traumatization) in a safe manner while contributing to the discussion in a meaningful way. It is refreshing to see learning from the initial workshop, clearly indicating problems that needed to be addressed, being discussed in a candid way. The point around 'standard university research ethics processes are ill-equipped to support adaption of standard research approaches to meet the needs of particular populations' is well taken. If possible, within the word limit, some elaboration of this would make this even more useful for the reader.	Thank you for your supportive comments. We have added some references to the literature about ethics processes on p.11 as there wasn't space to expand further.
2. It would also be useful for researchers planning workshops to keep in mind advantages and disadvantages when considering whether online forums or offline forums are most appropriate as well as consider hybrid formats while recognising the challenge of organising them and engaging both online and offline participants simultaneously.	Thank you for suggesting this. We have added the following to p.10: 'Hybrid workshops bring similar challenges in addition to supporting in-person attendees.'
**Reviewer 2**	
3. Many thanks for the invitation to review this manuscript. The authors present a thoughtfully	Thank you for the feedback.

	written account of challenges encountered during the conduct of a multi-phase consensus study with the overall aim of developing core outcome sets to enhance interventions for family violence and abuse. In the current paper, the investigators offer a reflective commentary on the challenges and adaptations made to foster a trauma-informed environment in multi-stakeholder workshops that included adults with lived experience of CM and DVA, alongside researchers and practitioners.<br><br>The manuscript is really well written, and the reflections are engaging. The authors make a compelling argument that standard approaches to public involvement must be adapted when conducting research in the intersecting and complex areas of CM and DVA. Their emphasis on ensuring that studies are not only safe but also positive and empowering for participants with lived experience underscores the need for innovative and iteratively refined methods. Funders and decision-makers should be made aware of the complexities and challenges inherent in this approach so that such research is adequately resourced, enabling genuine inclusion that moves beyond tokenism.<br><br>Without having read an original submission or previous reviewer comments, if any, I offer a few suggestions to strengthen the manuscript for publication.	
4.	Instead of presenting a formal thematic analysis, the authors provide informal reflections on the workshop process. While these reflections are insightful, a more structured qualitative analysis of the workshop data, as indicated in the previously published protocol (Howarth et al. 2022), would balance descriptions of what worked well with the challenges encountered. The protocol indicates a thematic analysis using Braun and Clarke; however, this analysis is not fully realised in the current commentary. A formal analysis could identify clear themes in participants' feedback, providing readers with a more complete understanding of the workshop dynamics. Further, incorporating quotes from participants would bring readers closer to the authentic voices of those involved. If the choice to report reflections in a commentary rather than perform thematic analysis was due to logistical constraints or other reasons, an explanation in the	Thank you for this suggestion. We chose to submit this as a 'communication article', the purpose of which is not to present primary research but to comment on the conduct of research: Introducing BMJ Open's new article type: The Communication Article - BMJ Open. We carried out the thematic analysis (mentioned in the protocol) on the workshop content focused on outcomes, the results of which are reported here: Child and family-focused interventions for child maltreatment and domestic abuse: development of core outcome sets | BMJ

	methods would provide important context for readers.<br><br>The manuscript focuses more on the challenges encountered in the conduct of the workshop and the subsequent pivot to implementing a more trauma-informed approach. A more complete analysis might have revealed some of the successful elements alongside difficulties and would present a comprehensive view of the adaptations.	Open. However, we did not capture workshop dynamics or feedback as this was not the aim of the research (and we weren't anticipating the challenges we faced) and thus we don't have any relevant quotes nor the ethical approval to report quotes.<br><br>We have added the following to the methods section on p.4 to make this clearer: 'We have reported these reflections as a commentary because we did not formally collect data related to the process of workshop delivery and what follows reflects discussion and learning from the research team and survivor advisory group.'
5.	Providing demographic data, such as age, gender, ethnicity, lived experience affiliation, and LGBTQI status of workshop participants, researchers and practitioners, would offer deeper context for understanding the diversity of participant perspectives. A demographic summary table would enhance the analysis of trauma-informed adaptations.	We didn't collect demographic data from the workshop participants because the aim was to generate ideas for outcomes (they were consultation workshops rather than research workshops). We do have participant data for the surveys and interviews which are reported in the paper mentioned above.
6.	A more detailed description of how "trauma-informed" was defined and operationalised would be helpful. The authors could discuss how they arrived at addressing specific behaviours, facilitation methods, and written guidelines for participant interaction that were subsequently implemented.	Thank you for this suggestion. We have added in our definition of trauma-informed on p.2: 'We understood trauma-informed to mean avoiding re-traumatising participants and prioritising safety and trust. (12)'<br>We have inserted the following on p.6 to connect what follows with the definition:<br>'to ensure safety, build trust and avoid re-traumatisation'
7.	The manuscript does not appear to include a specific explanation for the lack of inclusion of children <18 in the workshops, which was well-delineated in the published protocol.	Thank you for flagging this, we have added it to the participant section on p.4: 'We did not directly involve children and young people aged under 18

	years because of the potential safeguarding issues if they had recent experiences of violence and abuse and were not engaged with support services.'

VERSION 2 - REVIEW

Reviewer **2**

Name **Otterman, Gabriel**

Affiliation **Linköping University, Department of Biomedical and Clinical Sciences**

Date **03-Dec-2024**

COI

This revised manuscript reflects the authors' thorough responses to reviewer comments and has been substantially improved. The authors have provided adequate clarification of the manuscripts' submission as a commentary article, in line with their aim of reflecting on methodological iterations rather than conducting a formal analysis. The addition of a definition of trauma-informed consensus process is helpful for the reader. The explanation on the exclusion of children <18 as a stakeholder group in this process is also helpful.

Please note that the status of reference #25 (the published protocol) as 'under review' should be updated.

With the frank reflections on a real-world research process, the manuscript makes a significant contribution to the literature on trauma-informed methodologies and PPI in sensitive research areas.

VERSION 2 - AUTHOR RESPONSE

R#1<br>1. It was a pleasure to read this important piece dealing with how to ensure survivors of violence/maltreatment can participate in a consensus workshop with other stakeholders (where there is the possibility of facing conflict and the risk of re-traumatization) in a safe manner while contributing to the discussion in a meaningful way. It is refreshing to see	Thank you for this feedback. We have elaborated on university ethics committees as follows on p.11:<br>'Paternalistic approaches to research ethics such as judging participants as 'too vulnerable' to take part can prevent research with people who have experienced trauma; this in turn limits our knowledge of violence and abuse. [34] Topics which research ethics committees deem to be too sensitive are not regarded as such by

learning from the initial workshop, clearly indicating problems that needed to be addressed, being discussed in a candid way. The point around 'standard university research ethics processes are ill-equipped to support adaption of standard research approaches to meet the needs of particular populations' is well taken. If possible, within the word limit, some elaboration of this would make this even more useful for the reader.	research participants, [33] highlighting the importance of lived experience involvement throughout review processes. In addition, committees are less likely to consider the impact on researchers of working on sensitive topics. [30,35] Finally it has been repeatedly shown that research ethics committees are inconsistent in their requirements and judgements adding to confusion for researchers. [31–33]'
R#2<br><br>This revised manuscript reflects the authors' thorough responses to reviewer comments and has been substantially improved. The authors have provided adequate clarification of the manuscripts' submission as a commentary article, in line with their aim of reflecting on methodological iterations rather than conducting a formal analysis. The addition of a definition of trauma-informed consensus process is helpful for the reader. The explanation on the exclusion of children <18 as a stakeholder group in this process is also helpful.<br><br>Please note that the status of reference #25 (the published protocol) as 'under review' should be updated.<br><br>With the frank reflections on a real-world research process, the manuscript makes a significant contribution to the literature on trauma-informed methodologies and PPI in sensitive research areas.	Thank you for your feedback and for spotting the missing date. This has been added to the reference list.
